# A systematic review: the dimensions to evaluate health care performance and an implication during the pandemic

**DOI:** 10.1186/s12913-022-07863-0

**Published:** 2022-05-09

**Authors:** Faten Amer, Sahar Hammoud, Haitham Khatatbeh, Szimonetta Lohner, Imre Boncz, Dóra Endrei

**Affiliations:** 1 Doctoral School of Health Sciences, Faculty of Health Sciences, University of Pécs, Pécs, Hungary; 2 Faculty of Health Sciences, Institute for Health Insurance, University of Pécs, Pécs, Hungary; 3 Clinical Center of the University of Pécs, Medical School, Cochrane Hungary, University of Pécs, Pécs, Hungary

**Keywords:** Balanced scorecard, Performance, Indicators, Health, Hospital, Evaluation, Assessment, COVID-19

## Abstract

**Background:**

The balanced scorecard (BSC) has been implemented to evaluate the performance of health care organizations (HCOs). BSC proved to be effective in improving financial performance and patient satisfaction.

**Aim:**

This systematic review aims to identify all the perspectives, dimensions, and KPIs that are vital and most frequently used by health care managers in BSC implementations.

**Methods:**

This systematic review adheres to PRISMA guidelines. The PubMed, Embase, Cochrane, and Google Scholar databases and Google search engine were inspected to find all implementations of BSC at HCO. The risk of bias was assessed using the nonrandomized intervention studies (ROBINS-I) tool to evaluate the quality of observational and quasi-experimental studies and the Cochrane (RoB 2) tool for randomized controlled trials (RCTs).

**Results:**

There were 33 eligible studies, of which we identified 36 BSC implementations. The categorization and regrouping of the 797 KPIs resulted in 45 subdimensions. The reassembly of these subdimensions resulted in 13 major dimensions: financial, efficiency and effectiveness, availability and quality of supplies and services, managerial tasks, health care workers' (HCWs) scientific development error-free and safety, time, HCW-centeredness, patient-centeredness, technology, and information systems, community care and reputation, HCO building, and communication. On the other hand, this review detected that BSC design modification to include external and managerial perspectives was necessary for many BSC implementations.

**Conclusion:**

This review solves the KPI categorization dilemma. It also guides researchers and health care managers in choosing dimensions for future BSC implementations and performance evaluations in general. Consequently, dimension uniformity will improve the data sharing and comparability among studies. Additionally, despite the pandemic negatively influencing many dimensions, the researchers observed a lack of comprehensive HCO performance evaluations. In the same vein, although some resulting dimensions were assessed separately during the pandemic, other dimensions still lack investigation. Last, BSC dimensions may play an essential role in tackling the COVID-19 pandemic. However, further research is required to investigate the BSC implementation effect in mitigating the pandemic consequences on HCO.

**Supplementary Information:**

The online version contains supplementary material available at 10.1186/s12913-022-07863-0.

## Introduction

Evaluating the health care sector is quite challenging and complex. Unsatisfactory performance can result from long waiting times (WTs), inefficiency, dissatisfactory patients, and health care workers' (HCWs) burnout [1, 2]. Coronavirus disease 2019 (COVID-19) imposed further burdens on the health care system worldwide due to the limited capacity of hospital beds and the increased psychological stress of HCWs during the COVID-19 pandemic [3, 4]. There is still a lack of information that would help health care managers and policymakers in the era of COVID-19 to improve the delivery of health care quality and to learn for the future [5]. Higher pandemic burdens, such as HCW burnout and stress, will rise when health care organizations (HCO) lack plans and preparedness to strengthen their surge capacity and HCW resilience [6, 7].

Researchers have employed different tools for the performance evaluation (PE) of HCO. The most utilized PE tools were the International Organization for Standardization (ISO standards), Malcolm Baldrige National Excellence Model (MBNQA), European Foundation for Quality Management (EFQM) Excellence Model, Singapore Quality Award (SQA), Six Sigma, Data Envelopment Analysis (DEA), Pabon Lasso Model, and Balanced Scorecard (BSC) [8–12].

The World Health Organization (WHO) initiated the Performance Assessment Tool for Quality Improvement in Hospitals (PATH) in 2003. It aimed to develop a framework for the assessment of hospital performance. The resulting dimensions from this project were clinical effectiveness, efficiency, HCW orientation, responsive governance, safety, and patient-centeredness. However, studies have shown that there are still some gaps in this model and issues concerning the dimensions investigated [13, 14]. Additionally, the Organization for Economic Co-operation and Development (OECD) launched the Health Care Quality Indicator (HCQI) project in 2006; it aimed to develop key performance indicators (KPIs) to compare quality in health care at the international level and achieve international benchmarking. This project concluded that health care must be safe, effective, patient-centered, timely, efficient, equitable, acceptable, and accessible [15, 16].

Most of the abovementioned managerial tools mainly focused on the KPIs related to quality, efficiency, productivity, and timeliness dimensions [8–12, 17]. Each of these dimensions is considered a dimension at the internal perspective of the BSC, which consists of four perspectives: the internal process, customer, knowledge and growth perspectives, and financial perspectives [18]. Dimensions are described as collections of homogeneous or related KPIs. They are also referred to as diagnostic related groups (DRGs) [19], which have been proven to allow performance comparisons across hospitals and positively impact efficiency improvement [19].

The use of KPIs in the health care system before the pandemic has been beneficial for many reasons. First, the satisfaction rates of patients and HCWs were increased. Second, they lead to better efficiency, effectiveness, and financial performance and adapt to new technologies and ideas. Third, they lead to higher productivity and profitability [20–22]. In the pandemic, it is also crucial for HCO to track the performance of KPIs, which could draw faster attention to areas that require rapid responses and strengthening [6].

**Fig.1:**
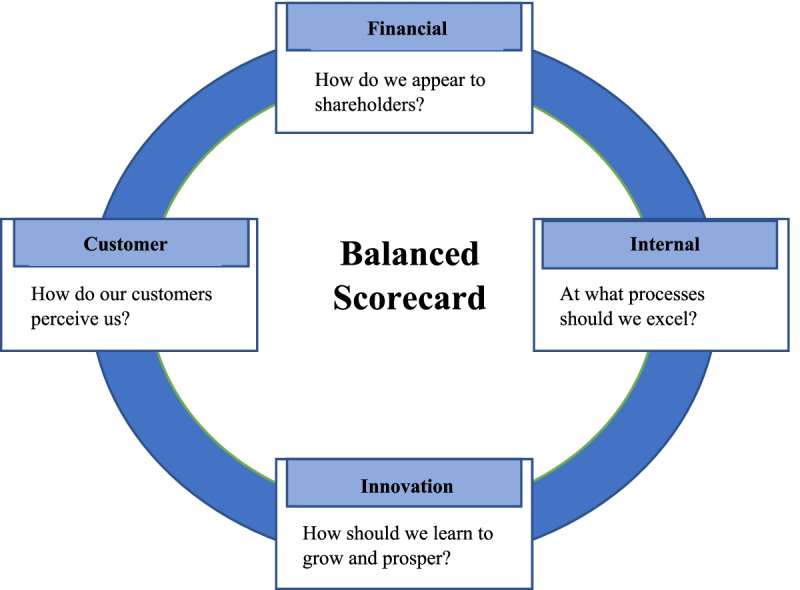
Balanced Scorecard Perspectives [18]

Most of the available PE models mainly focus on the internal perspective but lack coverage of the other dimensions or perspectives that are also important. BSC was considered different from the other managerial tools for two reasons. First, it offers a holistic approach to PE since it allows managers to highlight both financial and nonfinancial metrics. Second, the BSC is not only a planning or a PE tool. It is also a strategic managerial tool that assigns KPIs compatible with the HCO strategy [23, 24]. However, other PE tools, such as total quality management (TQM), lack these comprehensive properties [25]. The first generation of the BSC, unveiled by Kaplan and Norton in 1992, involved four perspectives: the financial, customer, internal process, and knowledge and growth perspectives, steered by the organizational vision and strategy [18]. See Fig. 1.

**Fig. 2:**
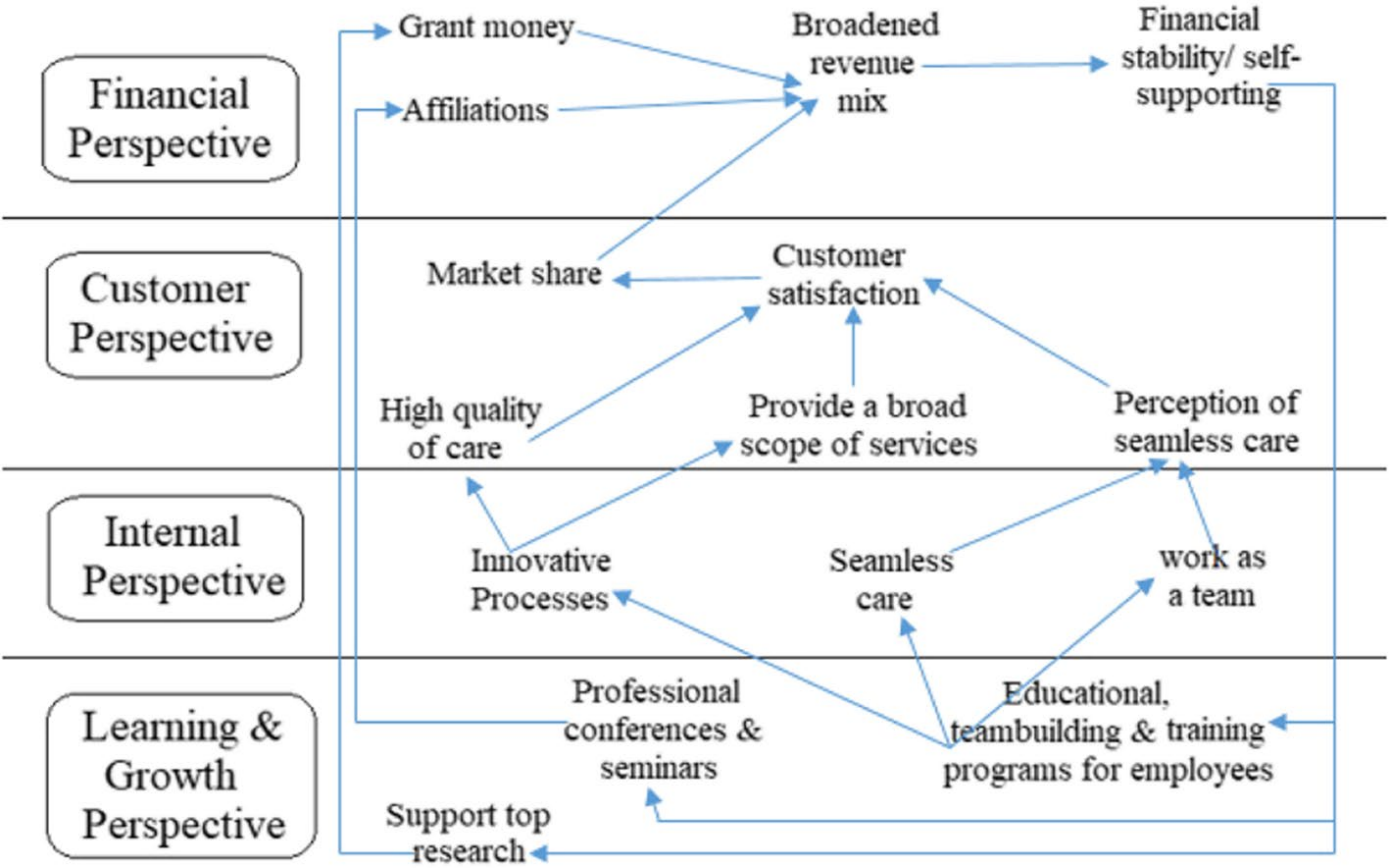
Duke University Health System Strategic Map [31]

Later, the second generation of BSCs was developed to include strategic maps, in which cause-effect cascades between perspectives or KPIs were inspected [23]. In the third generation of BSCs, a destination statement was incorporated, which evokes where the organization plans to go within a time horizon and the action plans to achieve each targeted objective [24]. In health care, Duke Children's Hospital in the United States of America (USA) was the first to implement the BSC in 1997. Figure 2 represents the strategic map of Duke University's health system. As a result, the hospital converted 11 million American dollar losses into four million profits after four years of implementation [25]. Since then, the BSC has gained increasing attention, and many HCOs in high-income countries and low- and middle-income countries have strategically utilized the BSC to develop their organizations [26–30].

A recent systematic review [32] proved that BSC implementations were effective in improving the financial performance of HCOs, elevating patient satisfaction rates, and to a lesser extent improving HCW satisfaction rates. Another review [26] revealed that there had been a lack of engaging stakeholders in BSC implementations, such as engaging patients and HCWs. However, researchers have pointed to the importance of patient and HCW engagement in the process of PE and delivery improvement [27–29]. The rest of the BSC reviews [26, 30, 33–42] focused only on the general narration of the BSC perspectives and subdimensions used. Moreover, none of them summarized the perspectives or dimensions of BSC based on their importance or frequency of use by health care managers. In other words, all the previous systematic reviews lack a systematic methodological categorization of perspectives, dimensions, and KPIs.

In correspondence with this research gap, this review aims at a) finding and recategorizing all the perspectives, dimensions, and KPIs that were employed in BSC implementations for unification purposes, b) ranking dimensions according to their frequency of use by HCO worldwide, and c) ranking dimensions according to their importance from the health care managers perspective.

## Methods

This systematic review is part of broad research. After assessing the impact of the BSC on stakeholder satisfaction [32] and before developing instruments to engage stakeholders in BSC implementations, we sought to accomplish the previously mentioned aims to summarize which dimensions were the most frequently used and essential as per health care managers in implementing the BSC. This review was conducted according to the 27-point checklist of the Preferred Reporting Items for Systematic Reviews and Meta-Analyses (PRISMA) checklist [43]; see Appendix (S1).

### Eligibility criteria

The inclusion and exclusion criteria were set as shown in Table 1 based on the PICO (Population, Intervention, Comparison, and Outcome) tool [44]. Additionally, all study designs were included.

**Table 1:** Inclusion/Exclusion Criteria and Search Strategy for PubMed

	**Inclusion criteria**	**Exclusion criteria**	**Search Strategy (MeSH terms and keywords) for PubMed**
Population	Health care organization which offers a primary, secondary, or tertiary health care or medical services such as (clinics, entire hospitals, or hospital's department), without restriction to the ownership or administrative type	Laboratories, pharmacies, biobanks, radiology departments, hospice homes and medical education centers, unless they were department or unit in the previously included institution types	hospitals[MeSH Terms] hospital department[MeSH Terms] health[MeSH Terms]
Intervention	Performance assessment of health care organizations through explicitly implementing BSC, or implicitly assessing the perspectives described in the initial BSC design [6]	Studies which explicitly used other TQM tools such as the MBNQA, ISO, SQA, six-sigma, etc	"quality indicators, health care"[MeSH Terms] scorecard*[Text Word] "score card*"[Text Word]
Outcome	Full reporting of indicators measurements or values	No reporting or partially reporting of indicators measurements or values	patient satisfaction[MeSH Terms] cost–benefit analysis[MeSH Terms] health care costs[MeSH Terms] Hospital personnel management[MeSH Terms] staff development[MeSH Terms] knowledge management[MeSH Terms] efficiency, organizational[MeSH Terms]
Study design	All study designs	_	No limitation regarding study design, type or time was set in the search strategy

*BSC* Balanced Scorecard, *TQM* Total Quality Management, *MBNQA* Malcolm Baldrige National Quality Award, *ISO* International Organization for Standardization, *SQA* Singapore Quality Award, *MeSH* Medical Subject Heading

### Data sources, search strategy, and study selection

First, a search strategy was developed for the PubMed database (see Table 1). Then, the strategy design was adapted for the Embase, Cochrane CENTRAL, and Google Scholar databases. Furthermore, an additional search in the Google engine was performed to find gray literature or unpublished papers, including theses and conference abstracts. Additionally, the reference lists of all the eligible articles were checked. The databases were searched from inception until October 2020.

The search strategy was developed by the first, second, and fourth authors; the first two were experts in health care management and BSC, while the fourth author was an expert in systematic reviews and meta-analysis. It was initially developed for the PubMed database. Moreover, the search strategy was developed to include both Medical Subject Headings (MeSH) terms and keywords to widen the search frame and obtain more results. Then, appropriate truncation and relevant indexing terms were used. See Appendix (S2), which shows search strategies in all databases. Afterward, the first author conducted an electronic database search and removed duplicates using the EndNote X9.2 program.

The selection of eligible studies was performed independently by the first and second authors in all steps. Disagreements were resolved by discussion after each step or, if necessary, through arbitration by the fourth author. First, the articles' titles and abstracts were examined to eliminate irrelevant papers between November 2020 and February 2021. Then, full texts were carefully inspected to decide the final papers' inclusion list between February and June 2021. If different KPIs were used in more than one implementation in the same study, each was counted as a different implementation. In comparison, implementations using the same KPIs in other locations or times in the same research were considered one implementation. The authors of studies with no available full texts or with partially reported results were contacted for missing data.

### Data extraction process

Data extraction was performed between June and July 2021 and then compared to discuss differences. The following data were extracted from the eligible studies: 1) author/s, 2) year of publication, 3) country of origin, 4) data collection duration, 5) data collection tool, 6) the number of perspectives, 7) the number of KPIs, 8) availability of weights/importance for perspectives or KPIs, and 9) outcome, which is represented in the KPIs that have been used and their weights/importance. The frequency of each KPI used at each implementation was plotted on Microsoft Excel, and the sum was calculated. In addition, the weight/importance assigned for each KPI at each implementation was reported on a scale of 100%. In the case of studies that did not give weights/importance explicitly, each KPI weight/importance was calculated by dividing one by the number of KPIs used in that study to assign an equal weight/importance for each KPI.

Consequently, we computed an average of the weights/importance assigned for each KPI. Next, we performed regrouping and coding for the KPIs to find the frequency of use and the set weights/importance percentages for each dimension. Then, the resulting major and subdimensions were listed and described between August and September 2021.

The research design of eligible studies was extracted directly from the studies. However, if the research design was not explicitly mentioned, we determined it based on the role of the investigator in that study. Specifically, the study was considered observational if the BSC exposures were naturally determined and the investigator had no part. On the other hand, the study was considered experimental if the investigator actively assigned the BSC intervention.

### Quality assessment

The risk of bias (RoB) assessment was accomplished between August and September 2021 to assess the quality of the included studies. The Risk of Bias in Nonrandomized Intervention Studies (ROBINS-I) tool was used to evaluate the observational and quasi-experimental studies [45]. In comparison, the Cochrane (RoB 2) tool was used for the assessment of randomized controlled trials (RCTs), as per the Cochrane collaboration's guidelines [46]. The RoB was analyzed at the study level and across studies since authors should avoid summarizing the overall RoB as per the Cochrane Handbook guidelines [47, 48].

In the RoB 2 tool, five types of bias were assessed: bias arising from the randomization processes, bias due to deviations from intended interventions, bias due to missing outcome data, bias in the measurement of outcomes, and bias in the selection of the reported results. On the other hand, in the ROBINS-I tool, seven types of bias were assessed: bias due to confounding, bias in the selection of participants in a study, bias in the measurement/classification of interventions/exposures, bias due to deviations from intended interventions/exposures, bias due to missing data, bias in the measurement of the outcomes, and bias in the selection of the reported results.

Using the RoB 2 tool, each type of bias was assessed as low, high, or unclear. While using the ROBINS-I tool, each type of bias was evaluated into five categories: low, moderate, serious, critical, or no information. Figures for RoB were prepared using the ROBVIS (Risk Of Bias VISualization) tool [49]. Last, it was recommended not to advocate quality appraisal as a criterion for inclusion in reviews [50]. Therefore, the authors decided to include all studies in this systematic review regardless of their quality assessment. See Quality assessment below.

## Results

### Study selection

A total of 4028 studies resulted from running the search strategy in the four databases. In addition, another three studies were identified through a Google search. Therefore, a total of 4031 studies were included. Duplicates were removed (*n* = 1046) using the EndNote program, and then the remaining articles were screened based on their titles and abstracts (*n* = 2985). Irrelevant papers were excluded (*n* = 2794).

**Fig. 3:**
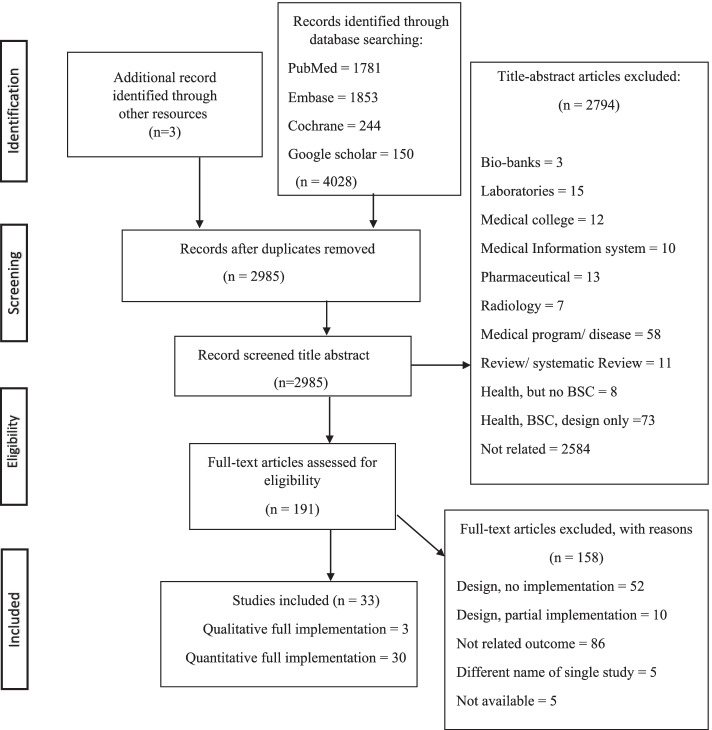
PRISMA Flow Diagram

Consequently, the remaining 191 studies were examined by reading the full texts. Among these papers, 22 papers were written in non-English languages, including Spanish, German, French, Chinese, and Persian. A full-text translation was performed for each study to decide whether to include or exclude any of them. As a result of reading the full texts, 158 studies were excluded, and only 33 were eligible for this review, in which 36 full implementations of different BSC designs were actually applied. Table 2 shows a summary of the 36 implementations. Details of the study selection process are shown in the PRISMA flowchart [43]. See Fig. 3.

**Table 2:** Overview of Included Studies

Author (s)	Year of publication	Country	Duration of data collection	Health organization type	Data collection tool	Number of perspectives	Number of indicators	Weight /importance (Yes/No)
Pink et al.[51]	2000	Canada	1997–1998	One hospital	Surveys + hospital reports	4	38	No
Zbinden et al. [52]	2002	Switzerland	April- October 2001	Three departments at a hospital	Personnel statistics and management system + annual reports + questionnaires + accounting system	4	18	Yes
Griffith & Alexander [53]	2002	The USA	1996–1998	2300 community hospitals	Medicare database	4	9	No
Biro et al. [54]	2003	The USA	1998–2001	63 centers and clinics	Chart audits + surveys + hospital data	5	17	Yes
Smith & Kim [55]	2005	The USA	2001–2004	Two departments in two hospitals	Survey + audit checklists	5	24	No
Devitt et al. [56]	2005	Canada	2004–2005	One hospital	Hospital records	5	26	No
Martinez-Pillado et. al. [57]	2006	Spain	2005	One hospital	Hospital records	5	32	No
Goodspeed [58]	2006	The USA	Jan-06	One hospital	NR	4	17	No
Yang & Tung [59]	2006	Taiwan	2000–2002	21 hospitals	Secondary data from the department of health + questionnaires	4	16	No
Chen et al. [60]	2006	China & Japan	In Japan (April 2003- March 2004). In China (January 2003-December 2003)	Two hospitals	Hospital measurement model	4	19	No
Peters et al. [61]	2007	Afghanistan	January-October 2004	617 health facility	National Health Services Performance Assessment + patient interviews + HCW & community members	6	29	No
Josey & Kim [62]	2008	The USA	Dec-06	One hospital	HCW satisfaction survey + Gallup for patient satisfaction	5	26	No
Chang et al. [63]	2008	Taiwan	2001–2005	One hospital	NR	5	12	No
Hansen et al. [64]	2008	Afghanistan	2004–2006	> 600 health facility	National Health Services Performance Assessment + patient and HCW interviews	6	29	No
Chu & Wang [65]	2009	Taiwan	2004–2006	One department at a hospital	Data extraction from hospital financial and performance records + questionnaire to director, assistant directors, head nurses & supervisors	4	11	Yes
Lupi et al. (1) [66]	2011	Italy	2007- 2009	One hospital unit	Data extraction from hospital records	4	26	Yes
Lupi et al. (2) [66]			2008–2009			4	34	Yes
Edward et al. [67]	2011	Afghanistan	2004–2008	615 health facilities	Performance Assessment + National Health Services patient and HCW interviews	6	29	No
Chen et al.[68]	2012	Taiwan	2004–2010	67 departments at a medical center	Secondary data collected by repeated measurements	4	9	Yes
Khan et al. [69]	2013	Bangladesh	January–February 2009	637 Health facilities	Questionnaire and exit interview questionnaire for clients	4	19	No
Lin et al. [70]	2013	China	July 2008-December 2009	One hospital unit	NR	4	32	Yes
Ajami et al. [71]	2013	Iran	NR	One hospital department	Top managers interview questionnaires + staff observations	4	20	No
Mutale et al. [72]	2014	Zambia	2011–2013	12 health facilities	HCW & patient Interviews + patient observations + households survey	7	20	No
Rowe et al. [73]	2014	Afghanistan	March- August 2010	24 health facilities	Patient-provider clinical interactions observations + Patient follow-up exit interviews + HCW interviews + facility record audits	5	26	No
Edward et al. (1) [74]	2015	Afghanistan	2012	One health facility	Quantitative and qualitative community survey	2	19	No
Edward et al. (2) [74]						2	16	No
Edward et al. (3) [74]						2	17	No
Rabbani et al. [75]	2015	Pakistan	2012	Six health centers	Survey + services assessment + patient questionnaire exits interviews + HCW questionnaire interview	5	20	No
Teklehaimanot et al. [76]	2016	Ethiopia	January – February 2010	433 health facilities	Structured & semi-structured internationally accepted questionnaires (health facility audit + HCW interviews, community interviews)	6	32	No
Catuogno et al. [77]	2017	Italy	2007–2008 & 2014 -2015	One department at a hospital	Stakeholder satisfaction questionnaires + hospital discharge report + charity report + departmental report + hospital discharge database	4	25	No
Gao et al. [78]	2018	China	NR	Five hospitals	HCW questionnaires + Patient interview-based questionnaire + TOPSIS method	4	36	Yes
Ebrahimpour et. Al [79]	2019	Iran	2010–2017	One hospital	Hospital records	4	23	No
Widyasari & Adi [80]	2019	Indonesia	During 2018	One hospital	structured interviews + semi-structured interviews + documentation + Observation	4	11	Yes
Mabuchi et al. [81]	2020	Nigeria	April–May, 2016	111 primary health facilities	Survey + interview questionnaire	6	32	No
Manolitzas et. al. [82]	2020	Greece	NR	One hospital department	Interviews + hospital records + observation	4	11	Yes
Gonzales et. al. [83]	2020	NR	NR (but data extracted 2018–2019	One medical center	Hospital records	8	13	No
						**Average 4.5**	**Average**	**Total**
							**22**	**Yes: 10**
								**No: 26**

*USA* United States of America, *HCW* Health Care Workers, *TOPSIS* Technique for Order of Preference by Similarity to Ideal Solution, *NR* Not Reported

### Study characteristics

#### Language and location

From the resulting 36 implementations, one was in Spanish [57], one was in Persian [79], and the rest were in English. The 36 implementations were performed in various countries: 19 in.

Asia [59–61, 64, 65, 67–71, 73–75, 78–80, 84], seven in North America [51, 53–56, 58, 62], six in Europe [52, 57, 66, 77, 82], three in Africa [72, 76, 81], and one without location information [83].

#### Settings

Twenty-one implementations were performed in hospitals (secondary and tertiary HCO) [41, 51–53, 55–62, 64, 66, 67, 71, 77, 79, 80, 82] and 15 in medical centers or health facilities (primary HCO) [54, 65, 68–70, 72–76, 81, 83, 84].

#### Implementations

Two studies [66, 74] included three and two implementations, respectively, with different KPIs per implementation. Thus, the 33 resulting studies contained 36 unique implementations. No BSC implementation in the COVID-19 era was found.

#### Study design

The 36 BSC implementations varied in their designs. However, most studies did not explicitly report their study design. We categorized the 36 implementations based on the active role of the investigator in BSC implementation and the time of data collection. Consequently, one sole study design was an RCT [72]. Moreover, 14 implementation designs were uncontrolled quasi-experiments. Specifically, six implementations had a posttest-only design [52, 60, 71, 78, 81, 82]. Five implementations in four studies had pretest–posttest designs [61, 66, 77, 79]. Finally, three implementations interrupted the time series design [54, 67, 85]. On the other hand, 20 implementations were observational; six implementations in four studies were cross-sectional [53, 73–75], one implementation was prospective [86], ten implementations were retrospective [58, 59, 62, 64, 65, 68–70, 76, 84], and two implementations were prospective and retrospective [55, 56]. Finally, one implementation did not have sufficient information or reported the study design [83].

### Decision model

Some of the resulting studies integrated multiple-criteria decision analysis (MCDA) with BSC. One study [82] combined BSC with simulation and MCDA techniques with what was referred to as S-MEDUTA. Another study [60] integrated the BSC with fuzzy analysis. Two studies [67, 78] combined BSC with AHP, and one [78] used the TOPSIS technique. Studies explained that using these methodologies with the BSC would help them arrive at more informed and better decisions.

### Perspectives frequency of use and importance

A total of 797 KPIs were extracted from the resulting implementations. These KPIs were categorized in the studies under 15 perspectives. The average number of perspectives used per study was 4.5, and for the KPIs, it was 22. The most frequently used perspectives were the internal, financial, patient, learning and growth, HCW, managerial, community, and stakeholder perspectives. The total use frequencies of these perspectives at the implementations were 29.6%, 17%, 12.6%, 12.6%, 9.4%, 6.3%, 5%, and 3.1%, respectively. On the other hand, the topmost important perspectives from the health managers' viewpoint were the internal, financial, learning and growth, patient, HCW, community, managerial, and stakeholder perspectives with a total weight/importance of 37.9%, 15.4%, 12%, 11.3%, 7.8%, 7.7%, 3.6%, and 2.8%, respectively.

### Categorization and regrouping of KPIs into Dimensions/Subdimensions

**Fig. 4:**
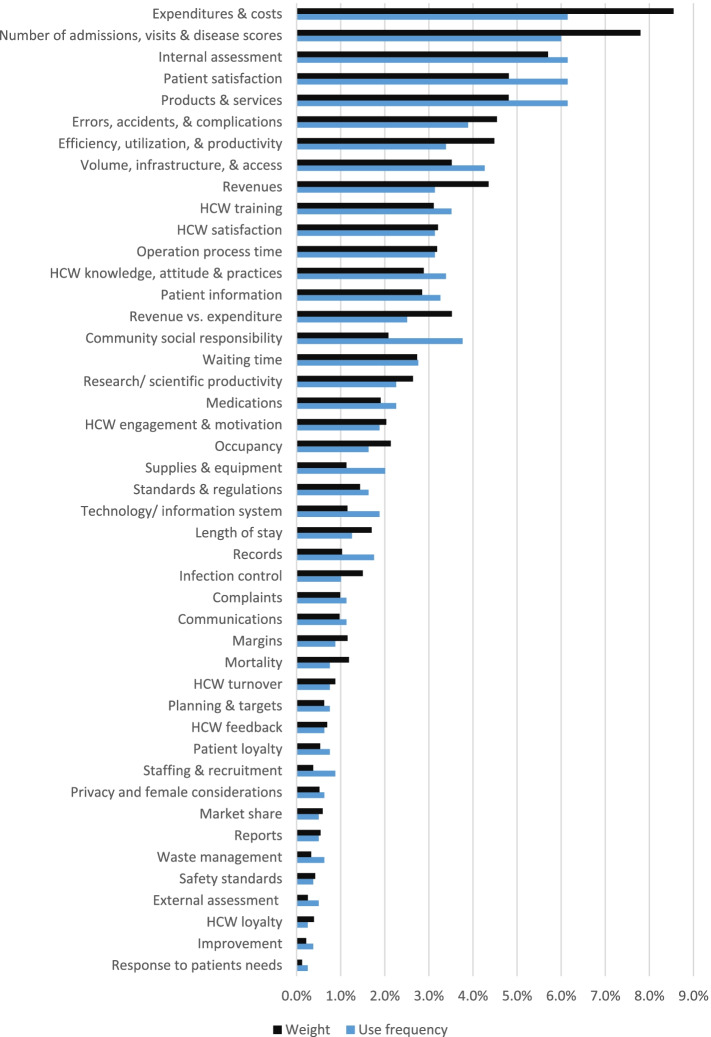
The BSC 45 subdimensions. Figure legend: After regrouping the 797 indicators, 45 subdimensions resulted. This figure shows the frequency and weight/importance for each subdimension

**Fig. 5:**
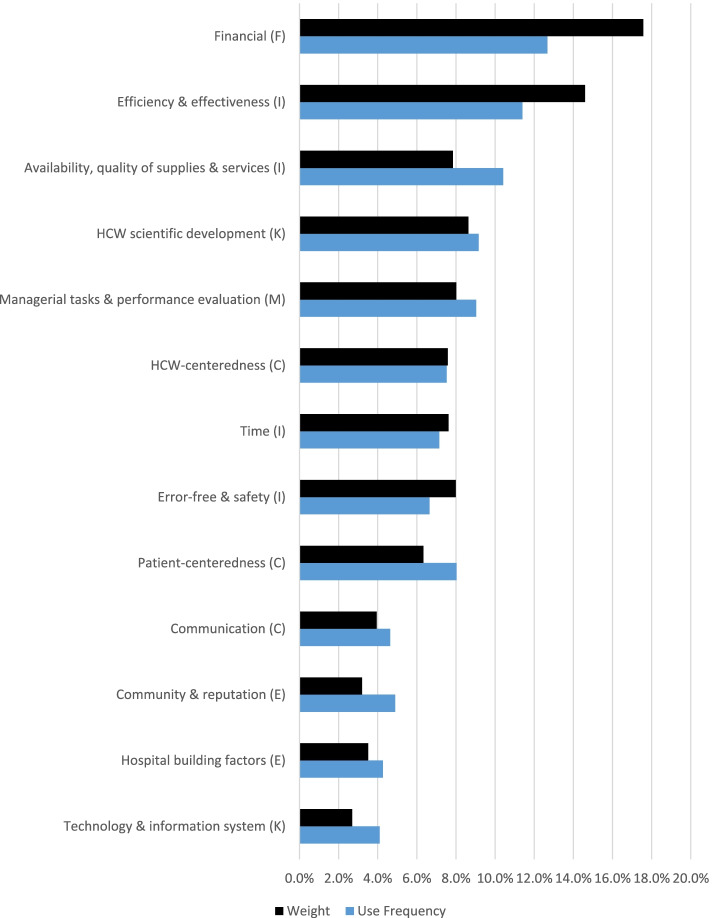
The BSC 13 major dimensions. Figure legend: Reassembling the 45 subdimensions resulted in 13 major dimensions. This figure shows the frequency and the weight/importance for each major dimension independently

The 797 extracted KPIs were plotted according to their frequencies and weights/importance in the categorization process. See Appendix (S3). Grouping and recategorizing KPIs resulted in Figs. 4 & 5 below, showing 13 major dimensions and 45 subdimensions based on their.

**Fig. 6:**
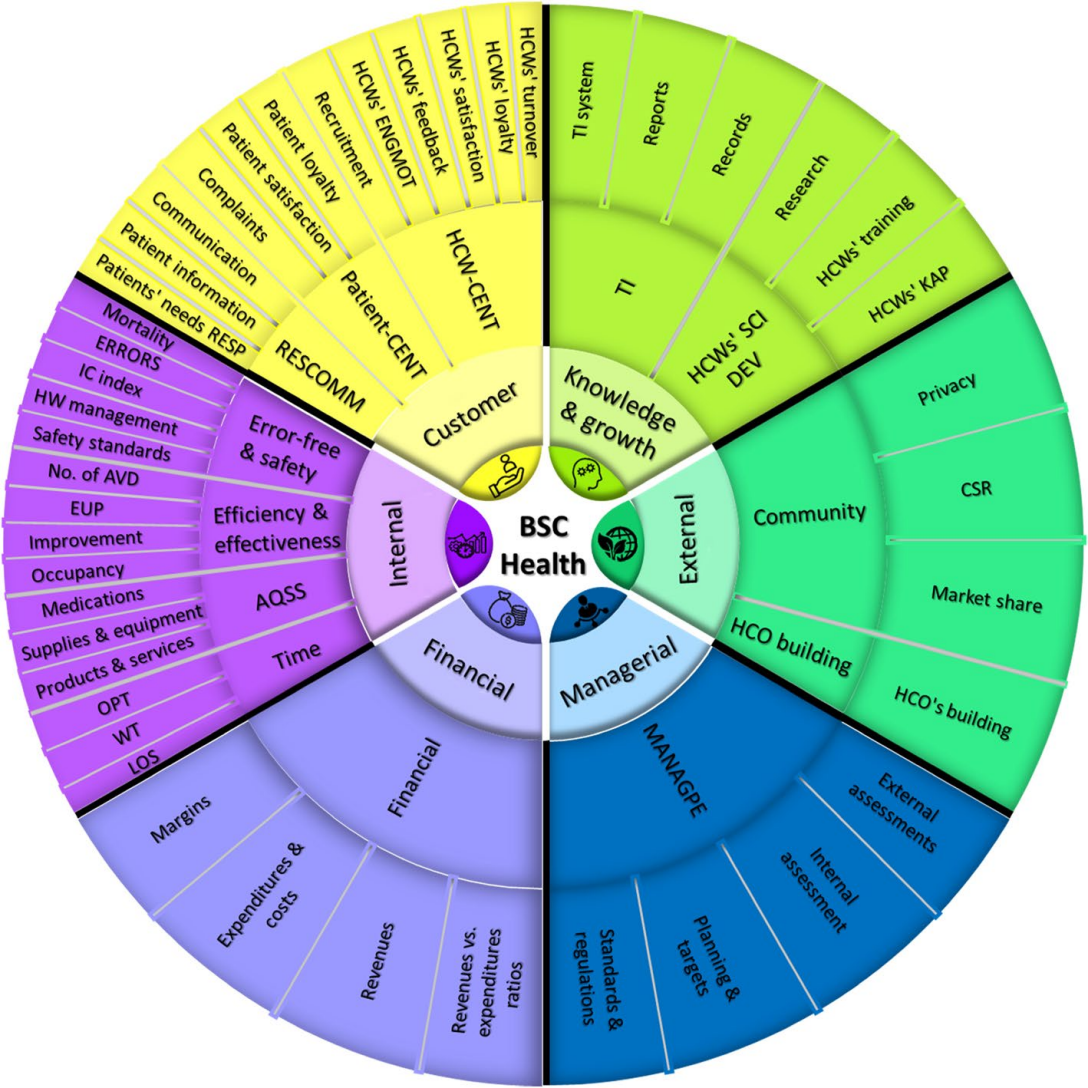
A summary of BSC perspectives in health care and their contents. Figure legend: Summary of BSC perspectives and the underlying major and minor subdimensions for the PE of HCOs

Frequency of use and importance, respectively. After regrouping these KPIs into homogenous major dimensions and subdimensions, 13 major dimensions resulted, with 45 subdimensions. A summary of the resulting perspectives and their major and subdimensions contents are illustrated in Fig. 6. The description of each major and subdimensions is described further in Appendix (S4).

### Quality assessment

Each study was evaluated in terms of RoB, as illustrated in Appendix (S5). The RoB 2 tool was utilized to assess the ROB in the sole RCT study [72], for which the assessment showed fair evaluation, except for performance bias. On the other hand, utilizing the ROBINS-I tool for assessing the RoB in observational and quasi-experimental studies revealed no information about confounders' adjustment methods except in three studies [51, 59, 67]. The confounding agents were apparent in the three studies; one study [51] performed confounders adjustments. On the other hand, another [59] adjusted for patient severity but not for the LOS and mortality rate. Last, one study [67] did not perform adjustment at all, which may have affected measurement precision.

The selection bias across studies reflected a serious RoB in five studies [59, 63, 67, 73, 80]. Therefore, the intervention and the follow-up did not coincide, and a potentially substantial amount of follow-up was missing in their analysis. Studies with a moderate risk of intervention/exposure measurement bias reflected a well-defined intervention status, but some aspects of the assignments of intervention status were determined retrospectively. Furthermore, bias in selecting the reported results was serious in one study that partially reported the results [80]. Studies that reported all results but did not have a preregistered protocol or whose outcome measurements were not defined in an initial plan were given a moderate risk. See Appendix (S5).

## Discussion

### Discussion of the main results

All the perspectives, dimensions, and KPIs employed in BSC implementations were collected to fulfill the research aims. Categorization and regrouping of the KPIs into major and subdimensions were performed. Then, the dimensions were ranked according to their frequency of use and their importance. The BSC tool can offer comprehensive planning, monitoring, evaluation, and improvement of HCO KPIs. Hence, their performance should be improved in the short and long term.

In general, studies had either no information or low or moderate ROB. At the same time, only a few of them had serious or critical ROB. However, studies that had only fully reported BSC indicator measurements were included. Many of them did not have a preregistered protocol or predefined measures in their plan. No information was found regarding confounders or deviation from unintended interventions.

### Overall completeness and applicability of evidence

Analyzing the results shows that BSC implementations typically utilized four fundamental perspectives: financial, customer, internal, and knowledge and growth. However, the analysis of Fig. 5 revealed the frequent employment and the importance of other BSC perspectives in BSC implementations. Specifically, the external and managerial perspectives. This reflects the need for slight modifications of BSC design and corresponds with the findings of another study [87], which referred to the sustainability perspective of the BSC as the fifth pillar. Additionally, our findings reveal that focusing on both internal and external customers from the customer perspective is essential.

The variation among BSC implementations in the categorization of the same KPIs reflects the need for data standardization. HCW training-related KPIs, for example, were categorized under the learning and growth perspective in almost half of the resulting studies [58–60, 62, 64–78, 84]. Meanwhile, the rest of the studies categorized them under the perspectives of HCW [72, 76], quality [54], service capacity, provision/service capacity [64, 68, 70, 73, 76, 84], and healthcare facility functionality [75]. These results are consistent with a study [5] that referred to the lack of defining measures and the lack of data standardization. The differences in categorization prove our assumptions in the calculation imprecision in the previous reviews. Specifically, in the use frequency or the importance of the perspectives and KPIs. Our systematic review solved this calculation bias by uniformly forming the 797 KPI categorizations. Regrouping similar or semisimilar KPIs under the same category resulted in more precise results. Unification of dimensions can guide uniform future implementations of PE or BSC at HCO, allowing data sharing and comparability. Dimension unification can be why our findings are different from another systematic review [88] that did not consider unifying the classification of KPIs. According to HCO management, the average LOS, HAIs, patient satisfaction, bed occupancy, and bed turnover rate were the most useful KPIs.

Analyzing the results also shows a lack of BSC utilization in HCO during the pandemic. Additionally, there has been a lack of studies comprehensively examining the impact of COVID-19 on KPIs.

This review can guide healthcare managers and researchers since the resulting dimensions can be utilized to synthesize future BSC measurements. Specifically, the dimensions can direct the creation of new instruments to engage stakeholders in future BSC implementations. Moreover, this review can provide a road map for healthcare managers to perform a comprehensive PE of HCO during the COVID-19 pandemic. Since the COVID-19 pandemic may influence the BSC dimensions positively or negatively compared to prepandemic, analyzing the effect of the pandemic on the performance of the major- and subdimensions will allow HCO’s managers to better understand where to focus on their action plans to improve the overall performance of HCOs.

### Practical assessment implications of the resulting dimensions in the COVID-19 era

Although this systematic review included ten months after the initiation of the COVID-19 pandemic, no research on BSC utilization in COVID-19 was found. Moreover, health policy experts stated that insufficient standardization of quality measurement approaches in the COVID-19 era challenged sharing purposes. As a result, the comparison between the performance of healthcare systems is disrupted [5]. Comparison is critical in cases where the optimal performance is not fully understood as in pandemics, and a comparison with other health systems would be informative and necessary [5]. Therefore, addressing the lack of data standardization was suggested to be overcomed by quickly defining measures, which could allow health systems, at least in the short term, to use standardized methods to better understand their performance [5].

We pursued further analysis in this paper based on independent studies per resulting dimension during the COVID-19 era to highlight how these dimensions can be utilized to monitor and improve HCO's performance during the COVID-19 pandemic.

#### The Financial major dimension

Due to COVID-19 hospitalizations at the beginning of the pandemic, health policy experts suggested that HCO in some regions will have more significant revenue and greater costs related to additional HCW and resources. In contrast, other hospitals will experience mostly sharp reductions in elective and outpatient payments, which will create unprecedented financial challenges for HCO [89]. However, in addition to the higher costs of HCWs and resources, researchers found higher costs of treatment due to extra diagnostic tests and isolation costs [90].

In the United Kingdom (UK), the total expenditure on the National Health System (NHS) has increased significantly during the pandemic [91]. The NHS made funding upgrades to expand waiting areas and treatment cubicles [92]. Some studies have focused on cost-effectiveness calculations. A study in South Africa indicated that purchasing intensive care unit (ICU) capacity from the private sector during COVID-19 surges may not be a cost-effective investment [93]. To date, there is still a lack of studies that handle the financial dimension or develop cost-saving strategies at the health organization level in COVID-19.

#### The efficiency and effectiveness major dimension

Analyzing the number of patient visits and admissions in the USA, [94] revealed a decrease in ER visits, increasing hospital admissions. However, another study in Alberta [95] perceived decreased admissions and ER visits to the hospital, despite the low volume of COVID-19 hospital admissions.

Many studies have been performed to analyze the efficiency, utilization, and productivity of HCO during the pandemic. A study [96] indicated that efficient hospitals under normal conditions lost their efficiency during COVID-19 and had to adapt to the new criteria. A systematic review [97] showed that healthcare utilization decreased by approximately one-third during the pandemic, with more significant reductions among people with less severe illnesses.

A study at an isolation hospital in Egypt [98] utilized the DEA tool to improve efficiency. This confirmed that the number of nurses and the number of beds impacted the operational efficiency of COVID-19, while the number of physicians had no significant effect on the efficiency. These results are compatible with a study in Mauritius [19] that found that nurses and beds are the most critical factors in hospital production; that is, a 1% increase in the number of beds and nurses resulted in an increase in hospital outputs by 0.73 and 0.51%, respectively.

#### The availability and quality of the supplies and services major dimensions

The supply and logistics management dimension was considered an important KPI in tackling COVID-19 [6]. This dimension includes evaluating the availability and quality of COVID-related medications, masks, personal protective equipment (PPE), detergents, medical services, supportive services, etc. Additionally, researchers viewed the availability of both clinical and supportive services at hospitals as essential in responding to the COVID-19 pandemic and the flow of COVID-positive patients [99]. The spectrum of supportive services to a hospital encompasses linen and laundry, diet, central sterile supply department, transport, consumables in large quantities at hospital stores, mortuary, and engineering services [99]. Some of the essential items were filtering face-piece respirators or N95 respirators and the availability of PPE kits [99]. The global challenge during this pandemic in terms of inadequate availability of PPE in HCO highlighted the vital role of CSSD. Centers for Disease Control and Prevention (CDC) suggested a method of decontamination, and reuse of filtering face respirators to overcome the shortage of these respirators is their extended use or reuse [99].

However, researchers have referred to the lack of studies on the quality of supplies and services at HCO in COVID-19 [5]. Lack of studies can be referred to as data lag in pandemics: the time between care provision and quality measurement reporting [5]. Policymakers suggested that measures should be less reliant on claims data, which by nature have a time lag, and focus on actions that can be generated from the electronic health record (EHR) [5] investigated during the pandemic.

#### The HCW scientific development major dimension

Due to its importance, many studies have aimed to evaluate HCW knowledge, attitudes, and practices (KAP) at the beginning of the pandemic [100]. HCW adherence to IC measures is affected by their KAP toward COVID-19 [101]. Some studies referred to insufficient knowledge about COVID-19 among nurses [102]. Surgeons were worried about losing their skills after months of lockdown due to paused practice [103]. However, HCWs were obliged to learn digital health skills and effectively communicate with patients during the pandemic [103].

A study [104] found that COVID-19 and non-COVID-19 publication productivity correlates with some factors. For example, epidemiologic, healthcare system-related, and pre-COVID publication expertise factors. Therefore, countries with a stable scientific infrastructure appear to maintain non-COVID-19 publication productivity nearly per year. More incentives must be drawn by HCOs to their HCWs to encourage research and scientific productivity related to COVID-19.

#### The managerial tasks and PE dimension

Standard policies, procedures, the availability of written standardized guidelines, and delivery in full and on time were considered essential in tackling COVID-19 [6]. A lack of standardization capability and conflicting or irrational managerial decisions were deemed dissatisfactory factors for HCWs in the pandemic [103].

Planning and preparedness are also crucial managerial tasks. The CDC developed a checklist to help hospitals assess and improve their preparedness for responding to COVID-19 [105]. Hospitals utilized a collection of some of the previously explained KPIs and dimensions to perform planning and internal assessment of their performance [106, 107].

Few studies [108, 109] have examined centralized governance's impact on HCO during the pandemic, which positively affected reactive strategies. Learning from past pandemics also positively influences proactive and reactive strategies. However, the role of PE internally, such as using BSC or MBNQA tools, or external assessments, such as Joint Commission International (JCI) accreditations, ISO certification, auditing, or peer review on HCO during the pandemic, still requires more investigation.

#### The HCW-centredness major dimension

Physicians referred to the importance of reliable acknowledgment and motivation both emotionally and financially, considering the sacrifices they provide every day [103]. In parallel, staffing and recruitment of an adequate number of medical and nonmedical HCWs were considered important KPIs for the PE of HCO at COVID-19 [6]. In the UK, the NHS employed strategies to facilitate the staffing process due to the shortage of HCWs. First, newly qualified/final year medicine and nursing students were deployed. Second, the return of the former HCW was made [110].

The HCW satisfaction rate and burnout have been evaluated in many studies during the pandemic. A study [103] showed that the physicians' burnout prevalence was 57.7% during the pandemic, which is considered high. HCWs who lack PPE reported lower occupational satisfaction than those who did not [103, 111]. HCW accomplishments during the pandemic were positively associated with higher occupational satisfaction rates [111]. Therefore, emphasizing HCW accomplishments leads to increased satisfaction rates.

Moreover, as mentioned earlier, better performance of the communication dimension, including psychological support, will raise HCW job satisfaction and lower the rates of burnout and stress [111, 112]. Some HCWs felt anxiety and fear mainly due to the possibility of transmitting the virus to their family members and the elderly living in their house [103]. A study in Canada [4] showed that HCW training and counseling services were perceived as helpful in reducing HCW stress. Despite that, they were underutilized in HCO.

On the other hand, although most nurses had to increase their workload due to staff shortages, a study [111] found that the elevation of the workload was not associated with lower occupational satisfaction. Additionally, another study in Singapore [113] found that HCW burnout was similar to the prepandemic rates. Nevertheless, the HCW vaccination, engagement, motivation, teamwork, and loyalty subdimensions and their impact are still not good.

#### The time major dimension

An "extra layer of processes" was added due to the donning and doffing protocols and cleaning requirements, which slowed all the operational processes down and increased the time required to accomplish serving the medical care to patients [91]. Patient WT was also influenced. In the UK, WT reached high levels in studies with a notable impact on elective surgery. The number of patients who waited for more than a year to receive NHS treatment in July 2020 was 81-fold greater than the previous year's number [92].

Moreover, the patient length of stage (LOS) also increased for another 2–3 days. A reason for this was the delays in COVID-19 testing results [114]. The LOS in the USA was two days more than that in Italy and five days less than that in Germany [115]. A systematic review for patient LOS in COVID-19 [116] concluded that LOS in China was longer than that in any other country, referring to differences in criteria for admission and discharge and different timings within the pandemic. Another study [105] found a negative association between the LOS and the case fatality rate. Therefore, LOS estimation can be introduced as a KPI to scale the success of the countries fighting the ongoing pandemic.

Moreover, LOS provides insights into when hospitals will reach capacity and predicts associated HCW or equipment requirements [116]. Discharge status should be considered when analyzing LOS since patients who are discharged alive have a longer LOS than those who died during their admission [116]. Hospitals reported that health insurance plans resisted paying for additional patient days in the hospital while awaiting COVID-19 test results [114].

However, complying with the CDC guidance on testing and disposition of patients was suggested to reduce the patient LOS, freeing up hospital beds for incoming COVID-19 patients [114]. Another study in the UK [114] indicated that due to the complexity and partiality of different data sources and the rapidly evolving nature of the COVID-19 pandemic, it is most recommended to use multiple LOS analysis method approaches on various datasets.

A combination of an accelerated failure time survival model and a truncation corrected method with the multistate survival model was found to be helpful in epidemic planning and management. Finally, the findings of a cohort study [117] concluded that a multimechanism approach effectively decreased the average LOS in the ICU by 5.4 days and up to nine days in older patients. This finding suggests that implementing this treatment protocol could allow a healthcare system to manage 60% more COVID-19 patients with the same number of ICU beds.

#### Major error-free and safety major dimension

This dimension includes monitoring, analyzing, and comparing mortality rates and investigating its determinants in HCO. Although mortality may not be directly related to errors, mortality rates higher than the average can reveal an underlying mistake. A cohort study in Mexico City [85] found that the mortality rates at the hospital's ICU and non-ICU departments were similar. The reason behind this finding was the ICU bed's unavailability. Approximately 45% of the patients who did not survive did not receive an ICU bed, which raised the mortality rate in the non-ICU admitted patients. However, this study revealed that the leading cause of non-ICU admission was acute respiratory distress syndrome. The leading cause of mortality for admitted patients was septic shock, followed by ARDS and multiorgan failure.

The WHO has provided clear guidelines for infection control (IC) during healthcare when COVID-19 is suspected or confirmed [118]. Patient safety was investigated in a systematic review of Indian-related studies [119]. Patient safety was negatively impacted during the COVID-19 pandemic due to inadequate preparation of the healthcare system, such as infrastructure and human and material resources. Additionally, researchers categorized diagnostic errors that could occur in the COVID-19 pandemic into eight types and suggested how to reduce them [120].

However, many studies have shown improvements in this dimension during the pandemic. A study in the UK [121] found a significant increase in the safety attitude questionnaire scores of doctors and other clinical HCWs and no change in the nursing group. It also showed a significant decrease in error reporting after the onset of the COVID-19 pandemic. Another study in Iran [122] found that health-associated infections (HAIs) during the pandemic were reduced, which could be referred to as the proper implementation of IC protocols. This finding is supported by a study in Ghana [123], which found that HCW compliance with IC measures was high during the pandemic.

The health waste (HW) management subdimension was intensively investigated due to the tremendous increase in HW volume during the pandemic [124]. A study in Iran [125] indicated that infectious waste increased by 121% compared with before the pandemic. Direct exposure of HCWs to virus-contaminated waste with inadequate safety measures and mismanagement of HWs may lead to their infection and facilitate the transmission of COVID-19 [124, 126]. The WHO has provided clear guidelines for managing healthcare waste during the pandemic [127]. Nevertheless, many studies worldwide [126, 128, 129] illustrated the existence of gaps and a flawed system for handling HWs during the pandemic.

A mini-review [124] of HWs during the pandemic showed that disinfecting waste, followed by proper segregation and on-site treatment, can also provide better and healthier HW management. It also revealed that surplus HW accommodation, mobile treatment, and temporary storage strategies might aid the sustainable management of healthcare waste without further spreading the virus. Another study in Brazil [128] proposed a model for the proper management of HWs. It focused not only on the operational management KPIs of HWs but also on environmental management, such as sustainable practices. Moreover, it highlighted the importance of employee training on HW guidelines since HW management was not considered an essential competence or a priority for every HCO.

#### The patient-centredness major dimension

Many studies have been conducted to evaluate patient satisfaction. A study [130] indicated no difference in patient satisfaction during the period spent in the emergency room before and during the pandemic. Another study [131] showed positive patient experience and satisfaction rates in Saudi Arabia's largest institutions during the pandemic. Moreover, many studies have focused on the psychological assessment of the impact of COVID-19 on the population in general. However, few studies have focused on specifically assessing the psychological effects on patients. For example, a study [132] found that COVID-19 patients with low education levels and females who have undergone divorce or bereavement tended to have a high prevalence of adverse psychological events. Another study [133] found that the psychological consequences of the pandemic were better handled by cancer patients 65 years of age or older, while younger cancer patients were more psychologically affected. Early psychological status identification and intervention should be conducted to avoid extreme events such as self-mutilating or suicidal impulsivity for patients [132]. Patient complaints and loyalty assessment during the pandemic and the psychological impact of COVID-19 on non-COVID-19 patients still need more investigation.

#### The responsiveness and communication major dimension

The main goal of HCO was considered to provide high-quality care to patients and meet their needs and expectations during an outbreak such as COVID-19 [96]. Moreover, dialog and listening to health demands in COVID-19-suspected patients was highlighted as the foremost step in the flows of care and guidance [134].

Communication among HCWs was also highlighted. A study [4] considered HCW reception of family support, colleagues, support, clear communication, and COVID-19 information as the most valuable resources in the pandemic. Lower HCW psychological distress symptoms, burnout, and intentions to quit were perceived when these communication resources were more available. Another study [112] indicated that gratitude in communication could reduce depression in HCWs by promoting social support and hope.

In addition, communication between HCWs and patients was also investigated during the pandemic. A study in Jordan [135] found that physician–patient communication positively affected the patient's psychological status in COVID-19. It recommended avoiding communication errors using jargon, not being available to patients, and not showing empathy in communication. Additionally, it emphasized the benefit of physicians as excellent listeners to patients. However, HCW-patient communication faced few obstacles during the pandemic. The protective equipment used by HCWs in the pandemic could have imposed a barrier to effective communication or eye contact with them [136]. Some pediatricians reported difficulty communicating with families and following up with patients, especially newly discharged neonates and infants, using the telephone [103].

However, more research is still needed to improve and evaluate patient education programs, patient guidelines, counseling and consultation services, and communication skills between HCWs and patients during the pandemic.

#### Community care and reputation major dimension

The external major dimension, including the social sustainability indicators for HCOs facing a crisis, can be ambiguous to define and apply, so SSIs have been organized under the broad categorical concerns of well-being, values, agency, and inequality [107]. Despite the doctor–patient confidentiality clause and the protection law for patient data privacy, the Department of Health and Social Care for England has relaxed the rules on sharing confidential patient data. It required HCO and the NHS to exchange patient information to help fight COVID-19 [137]. Moreover, COVID-19 patient data led to society breaching patient privacy in some countries [103, 138], which may have stigmatized those patients [103].

As mentioned earlier, a study [128] emphasized the importance of sustainable environmental practices for better HW management. The political situation was also considered an external influence during the pandemic. It was highlighted in a study [139] in the Palestinian territories, which referred to the COVID-19 situation in the presence of the Israeli military occupation to have a double epidemic effect, which eventually impacted the performance of the Palestinian health system HCO during the pandemic.

However, factors as exemptions offered by HCO for poor patients, social responsibility, patient privacy concerns, and HCO market shares in COVID-19 are still poorly investigated.

#### The HCO building major dimension

Design and infrastructure preparation were considered essential dimensions in some HCOs during the pandemic [6]. Healthcare systems made adaptations in HCO buildings after the COVID-19 pandemic. Examples include expanding waiting areas, increasing ICU capacity, establishing isolation areas, and building new hospitals [92]. In the UK, the NHS temporarily used private hospitals to provide public care, increasing the number of beds, ventilators, and all HCW categories. Moreover, nonhospital sites were temporarily turned into hospitals [110]. However, researchers did not sufficiently investigate the ease of access to HCO during the pandemic.

#### The technology and information system major dimension

Experts emphasized the role of technology and information (TI) in tackling COVID-19 as inevitable due to its importance in the response, prevention, preparedness, and recovery phases [140, 141]. TI system application varies from allowing HCO to maintain and share studies to producing different reports and follow-up with pandemic analysis. Telehealth is another example that proved helpful in the pandemic. It allowed HCWs to provide care for patients without direct physical contact, especially to patients at quarantine, while keeping them safe [142].

Researchers summarized the emerging technologies used to mitigate the threats of COVID-19 in the following categories: artificial intelligence/deep learning, big data analytics, high-performance computing infrastructures, robots, 3D printing technology, digital contact tracing technology, blockchain [113], bioinformatics systems, telemedicine, mobile phone, decision support system, the IC system in HCO, online interactive dashboard/geographic information system, Internet of Things, virtual reality, surveillance systems, and internet search queries [140, 141]

Governments, healthcare systems, and HCO need to keep updated with the emerging technologies in this field, allocate resources to invest in them, and develop the required skills in HCWs to utilize them properly.

### Strengths and weaknesses

We believe that this paper has several strengths. First, this systematic review includes all types of studies with BSC implementations, such as books, theses, conference papers, and letters to the editor. Second, this review contains all implementations despite the country, language, or HCO administrative type, which gives an advantage of generalizing HCO results worldwide. Third, unlike other BSC reviews [35, 37], which included definitions of biobanks, pharmacies, laboratories, radiology, and medical colleges in HCO, this review limited the report to primary, secondary, or tertiary healthcare organizations. However, an initial assessment by top management to evaluate the importance of each dimension and KPI based on the health organizations' strategy could be needed. This strategy leads to the homogeneity of the resulting studies and leads to more valid comparisons among the results. Fourth, this review calculates the use frequency of perspectives and the weights/importance assigned to them. Fifth, the first review has uniform KPIs in homogenous major dimensions and subdimensions despite the categorization differences among implementations, yielding more precise results. The resulting KPIs and dimensions in this review can be generalized or replicable to other HCOs and hospitals. Finally, this study is the first to analyze the implications of BSCs in HCO during the pandemic based on the literature. This implication provides a guide for future theoretical implications, such as performing systematic reviews for each major dimension during the pandemic. It also provides a guide for practical implications of BSC dimensions to assess HCOs’ performance.

On the other hand, this systematic review has some limitations. First, unlike previous studies, it excludes some HCOs, such as laboratories, pharmacies, radiology departments, and biobanks, as specified in the inclusion/exclusion criteria. Therefore, our results cannot be generalized to such HCO types. However, we excluded them to arrive at more homogenous KPIs and dimensions that are directly related to HCO that offer primary, secondary, and tertiary medical services. Second, it includes only the articles that report the complete implementations of BSC while excluding studies that display only the BSC design without reporting the full implementation results. Third, we extracted the KPIs from all resulting implementations despite their RoB. However, we included an ROB assessment for each implementation.

## Conclusion

In conclusion, our review shows that the most frequently used perspectives in BSC papers were internal, financial, patient, learning and growth, HCW, managerial, community, and stakeholder perspectives. The perspectives that had the highest importance were internal, financial, learning and growth, patient, HCW, community, managerial, and stakeholder.

Moreover, this review solves the dilemma of the KPI categorization difference between BSC implementations by dimension unification into 13 major dimensions. The financial, efficiency and effectiveness, availability and quality of supplies and services, managerial tasks, HCW scientific development, error-free and safety, time, HCW-centeredness, patient-centeredness, technology and information system, community care and reputation, HCO building, and communication. The proper utilization of the 13 major dimensions and the 45 subdimensions will serve as a planning, monitoring, evaluation, and continuous improvement tool for HCO, resulting in performance augmentation.

This research showed a lack of BSC utilization and any holistic PE approach in HCO during the COVID-19 pandemic. Additionally, some dimensions that are essential for PE are still poorly investigated. Our analysis reflects that most KPIs were negatively affected during the pandemic, except IC and safety measures, which improved in some cases. However, a comprehensive PE of HCO during the COVID-19 pandemic worldwide is still needed. Therefore, we recommend that future researchers perform a comprehensive practical PE for HCO during COVID-19 using the measurements of the resulting dimensions. This analysis will provide a better understanding of the causal relationships between dimensions. It will also allow comparability of the interventions' outcomes, which will boost the performance and mitigate the consequences of the pandemic on HCO. Moreover, researchers are encouraged to perform systematic reviews for each dimension, especially those that are already well investigated and the investigation of dimensions that are still poorly investigated but essential for PE. This theoretical implication will lead to performance enhancement and mitigate the consequences of the pandemic on HCO.

## Supplementary Information

**Additional file 1.**

**Additional file 2.**

**Additional file 3.**

**Additional file 4.**

**Additional file 5.**

## Data Availability

All data generated or analyzed during this study are included in this published article [and its supplementary information files].

## Abbreviations

BSC: Balanced Scorecard
CDC: Centers for Disease Control and Prevention
COVID-19: Coronavirus Disease 2019
DEA: Data Envelopment Analysis
DRG: Diagnostic Related Groups
EFQM: European Foundation for Quality Management
ER: Emergency Room
HAIs: Health-Associated Infections
HCO: Health Care Organizations
HCQI: Health Care Quality Indicator
HCW: Health Care Workers
HMIS: Health Management Information System
HW: Health Waste
IC: Infection Control
ICU: Intensive Care Unit
ISO: International Organization for Standardization
JCI: Joint Commission International
KAP: Knowledge, Attitude, and Practices
KPIs: Key Performance Indicators
LOS: Length Of Stay
MBNQA: Malcolm Baldrige National excellence model
MBO: Management by Objectives
MCDA: Multiple-criteria decision analysis
MeSH: Medical Subject Headings
NHS: National Health System
OECD: Organization for Economic Co-operation and Development
PATH: Performance Assessment Tool for quality improvement in hospital
PE: Performance Evaluation
PPE: Personal protective equipment
PRISMA: Preferred Reporting Items for Systematic Reviews and Meta-Analyses
ROA: Return on Assets
RoB: The Risk of Bias
ROI: Return On Investment
SAQ: Safety Attitude Questionnaire
SQA: Singapore Quality Award
T.I.: Technology and information
TQM: Total Quality Management
UK: United Kingdom
USA: United States of America
WHO: World Health Organization
WT: Waiting Time
