# Supplementary material for: A systematic review: the dimensions to evaluate health care performance and an implication during the pandemic
Source: BMC Health Serv Res. 2022 May 9;22:621. doi: 10.1186/s12913-022-07863-0 (PMC9081670; doi:10.1186/s12913-022-07863-0)
Supplement: Supplementary file 4 — Additional file 4. [file 12913_2022_7863_MOESM4_ESM.docx]

| **Table (3): Description of the BSC Major- and Subdimensions** | | | |
| --- | --- | --- | --- |
|  | **The major-dimensions** | **Description** | **Description of subdimensions and their KPIs:** |
| 1 | **Financial dimension** | Represented as the financial perspective in BSC.  It consisted mainly of four subdimensions. | **The first subdimension:**  Margins such as the cash flow margin and the operating profit margin.  **The second subdimension:**  Expenditures and costs such as personnel costs, controllable costs, and the cost per case or admission.  **The third subdimension:**  Revenues as the revenue per admission and the return per employee.  **The fourth subdimension:**  Revenues versus expenditures ratios, such as, ROI, ROA, and capital turnover. |
| 2 | **Error-free and safety dimension** | Part of the internal perspective of BSC. It composed of five subdimensions | **The first subdimension:**  Mortality, such as net death rate per 1000 patients or gross mortality.  **The second subdimension:**  Errors, accidents and complications subdimension, which contained KPIs such as complications index, hospitalized accident rate, medication error rate, blood preparation error, pneumonia complications.  **The third subdimension:**  IC index such as postoperative infection rate and infection prevention.  **The fourth subdimension:**  HW management includes segregating waste into proper sharps, infectious, pathological, pharmaceutical, radioactive and nonhazardous waste disposals. As well as waste minimization, color-coding, labeling, handling, transports, storage, treatment and disposal.  **The fifth subdimension:**  Safety standards focused on patient safety through the appropriate efforts to avoid adverse events related to errors in diagnosis, medication, or treatment, such as the full implementation of the 27 Safe Practices for Better Health care standards and percentage implementation of the National Patient Safety Goals. |
| 3 | **Efficiency and effectiveness dimension** | Part of the internal perspective. Through this dimension, four subdimensions are mainly focused on. | **The first subdimension:**  Number of admissions, visits and diseases; which evaluated the number of patients, surgeries, admissions, readmissions, cross-appointments, and disease scores.  **The second subdimension:**  Efficiency, utilization, and productivity included KPIs such as the case mix index, productivity percent, service utilization, ER patients per year per doctor, admitted inpatients per year and doctor, bed turnover rate, and nurses' workload.  **The third subdimension:**  The improvement subdimension, such as the cure, recovery, and improvement rates.  **The fourth subdimension:**  Occupancy, which mainly focused on bed occupancy rate, indicates the percentage of beds occupied by patients in a given period |
| 4 | **Availability and quality of supplies and services dimension** | Part of the BSC internal perspective. Under this dimension, three subdimensions are mainly focused on. | **The first subdimension:**  Medications, which included drug management, drug availability index, and tracer drug index.  **The second subdimension:**  Supplies and equipment, such as supply distribution system and equipment functionality index.  **The third subdimension:**  Products and services, this subdimension included KPIs, which evaluate either the variety of medical services or products offered by the HCO or their quality. |
| 5 | **Time dimension** | Part of the BSC internal perspective. It composed of three subdimensions. | **The first subdimension:**  Operation processing time is the time needed from the initiation of service until completion, for example, billing time, treatment time, correcting mistakes time, retrieving an archived file time, etc.  **The second subdimension:**  WT or the delay time until providing services is initiated.  **The third subdimension:**  LOS of an admitted patient till discharge. |
| 6 | **HCO building dimension** | It is usually part of the internal or the customer perspectives.  It included one subdimensions | Composed of one subdimension, it included KPIs which are related to HCO's building. For example, HCO's capacity, ER volume, waiting area, bathroom, cleanliness, water, electricity, appointments, ease of access, and ambulance availability. |
| 7 | **The responsiveness and communication dimension** | It is usually part of the customer perspective in BSC.  It composed of three subdimensions. | **The first subdimension:** Response to patients' needs, including response to their inquiries and feedback. This was performed either after patient arrival, during the admission and the treatment process, or before the discharge.  **The second subdimension:**  Patient information including patient information, education, guidelines, counseling and consultation services.  **The third subdimension:**  Communication which included evaluation for the nature of both internal and external communications, for example, the ability of coordination and teamwork among HCWs, and the relationships between HCWs and patients. |
| 8 | **Patient-centeredness dimension** | It is usually evaluated as a part of BSC customer or the stakeholder perspectives.  It included focusing on three subdimensions. | **The first subdimension:**  Complaints which mainly concerned with measuring the patients' complaint rate.  **The second subdimension:**  Patient satisfaction focused on measuring patient satisfaction rate in general or the satisfaction rate in specific toward a medical service, a specific HCWs dimension, or in a particular HCO department.  **The third subdimension:**  Patient loyalty is usually measured by patient retention and recommendations for that HCO. |
| 9 | **The HCW-centeredness dimension** | Either a part of the customer or the internal perspectives at BSC, while some studies added it under HCWs management perspective [30,48,55,62,67,72]. It consisted of six subdimensions. | **The first subdimension:**  Staffing and recruitment process starts from employment to the introduction process of the new employees.  **The second subdimension:**  HCWs' engagement and motivation include engaging doctors and nurses in the HCO managerial decisions and plans, high-performing HCWs rewarding, upgrade rate on the career ladder, involvement in bonus decision, and evaluation of HCWs motivation and burnout rates.  **The third subdimension:**  HCWs' feedback, for example, HCWs perception index surveys.  **The fourth subdimension:**  HCWs' satisfaction by doctors', nurses', other HCWs' satisfaction rates evaluation.  **The fifth subdimension:**  HCWs' loyalty index includes the doctors' and nurses' willingness to stay at the same HCO for another five years and recommend their colleagues to work at their HCO.  **The sixth subdimension:**  HCWs' turnover which assessed the number of doctors, nurses, etc., who left their jobs at that HCO in a specific period. |
| 10 | **The HCWs' scientific development dimension** | Part of the innovation and knowledge, or the customer perspectives.  It composed of three subdimensions. | **The first subdimension:**  HCWs' KAP, which concentrated on the HCWs' current competencies and knowledge in different medical and health related fields, the percentage of skillful employees, and their attitudes, behavior, and punctuality.  **The second subdimension:**  HCWs' training, through which the number of seminars, courses performed to improve the HCWs' KAP and the budget specified for this purpose were evaluated.  **The third subdimension:**  Research and scientific productivity, which can be a result when the previous two subdimensions are improved. For example, impact factor per HCW, total research impact of the HCO, number of participations in conferences and research programs per year, and the expenditure on the medical research. |
| 11 | **The technology and information system dimension** | Part of the innovation and knowledge perspective.  It consists of three subdimensions. | **The first subdimension:**  Records, such as: patient record index, HMIS records, hospital laboratory registrations, and medical records completion rate.  **The second subdimension:**  Reports, which included the ability to produce reports for different purposes in different HCO departments.  **The third subdimension:**  TI system, which was reflected by assessing the HMIS effectiveness, and the intensity of information use. |
| 12 | **The community and reputation dimension** | Part of the customer perspective, while some studies [48,49,69,72,78] added it under the community and social perspective.  It consists of three subdimensions. | **The first subdimension:**  Market share evaluation for the HCO in general or for a specific department at that HCO.  **The second subdimension:**  The CSR focused on the exemptions offered by the HCO for poor patients, benefits provided to the community, teaching and training programs offered for medical students, and the community satisfaction rate.  **The third subdimension:**  Privacy and female considerations evaluated the percentage of female patients, the availability of female doctors and nurses in the HCO, and the patients' privacy adherence. Although the female consideration in HCOs may not have significant importance in all cultures. However, it was vitally considered in some implementations [56, 57, 62, 66, 68]. |
| 13 | **The managerial tasks and PE dimension** | Part of the internal perspective. However, some studies added it under the managerial perspective [72,73].  It constitutes four subdimensions. | **The first subdimension:**  Standards and regulations, which can be reflected by the standardization capability for different HCO working processes. Moreover, the HCWs' awareness of these standards and regulations highlights the importance of the rules and standards to be clear, understandable, and specific to them.  **The second subdimension:**  Planning and targets subdimension incorporated business plan and target setting, updating, and HCWs' awareness and attention to them.    **The third subdimension:**  Internal assessment subdimension using managerial quality tools for quality enhancement, managing objectives, or the PE. For example, the included studies assessed the implementation of a continuous quality improvement system, MBO, TQIP, QOPI, and BSC, or the scores resulted from using different instruments and scales such as Press Ganey. This dimension also includes the internal PE process, such as regular performance review meetings, the visualization of performance data, and the HCWs' attention to performance.  **The fourth subdimension:**  External assessments including the accreditation, peer-reviews, and certificates the HCO receives from external sources. It includes evaluating the accreditation status of the HCO, such as JCI or AACI, plans to maintain it, and the performed periodic revising for the accreditation manuals. It also includes the certificates the HCO received, such as the ISO certificate and the peer reviews. |

BSC, Balanced Scorecard; KPIs, Key Performance Indicators; ROI, Return on Investment; ROA, Return on Assets; IC: Infection Control; HW, Health Waste; ER, Emergency Room; WT, Waiting Time; LOS, Length of Stay; HCO, Health Care Organization; HCWs, Health Care Workers; KAP, Knowledge, Attitude, and Practices; HMIS, Health Management Information System; TI, Technology and information; CSR, Community Social Responsibility; PE, Performance Evaluation, MBO, Management by Objectives; TQIP, Trauma Quality Improvement Program; QOPI, Quality Oncology Practice Initiative; JCI, [Joint Commission International](https://en.wikipedia.org/wiki/Joint_Commission_International); AACI, American Accreditation Commission International; ISO, International Organization for Standardization;
