# Supplementary material for: A systematic review: the dimensions to evaluate health care performance and an implication during the pandemic
Source: BMC Health Serv Res. 2022 May 9;22:621. doi: 10.1186/s12913-022-07863-0 (PMC9081670; doi:10.1186/s12913-022-07863-0)

Risk of Bias using ROBINS-I for Non-Randomized Studies of Interventions (NRSI) studies (Quasi- experimental and obdervational):


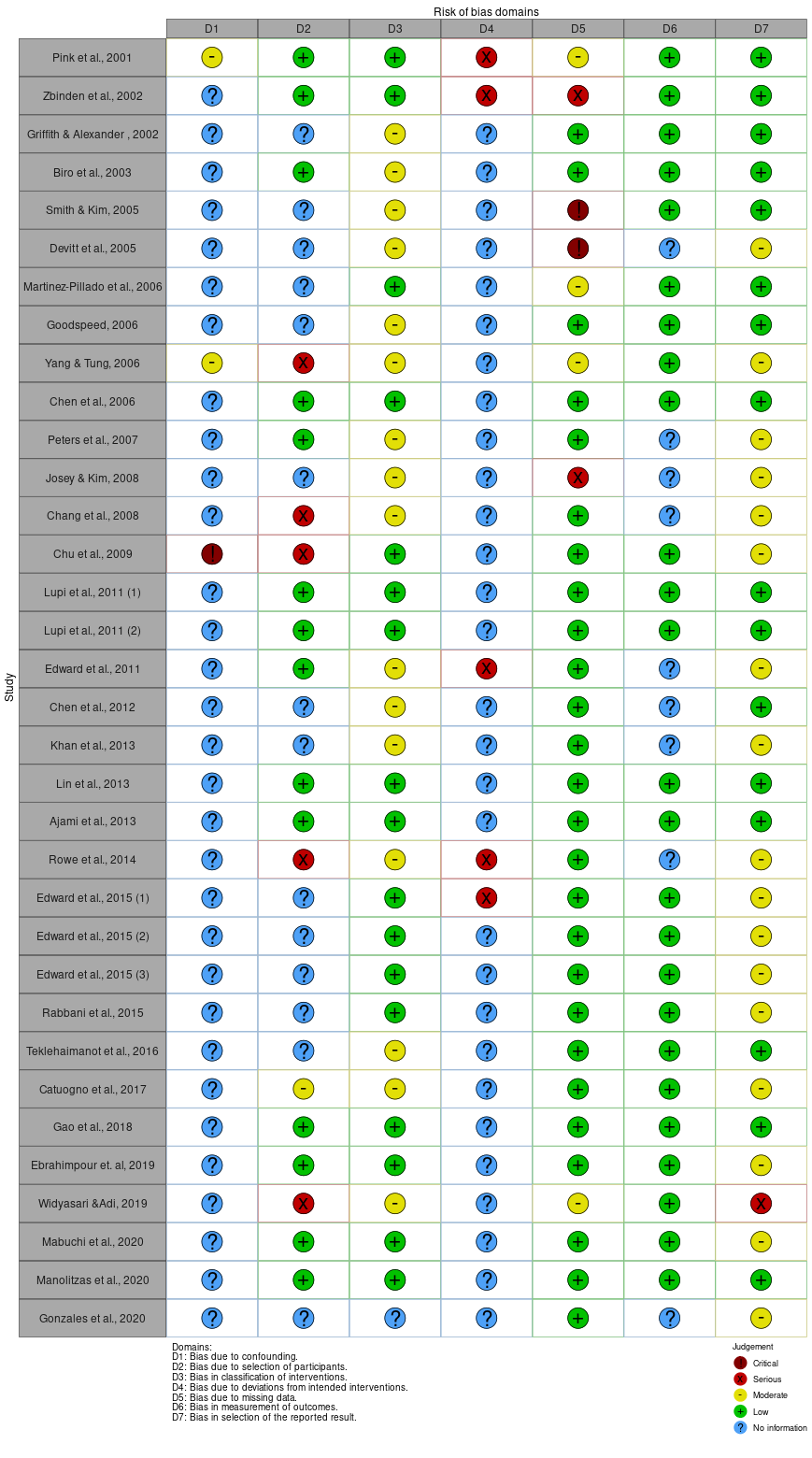


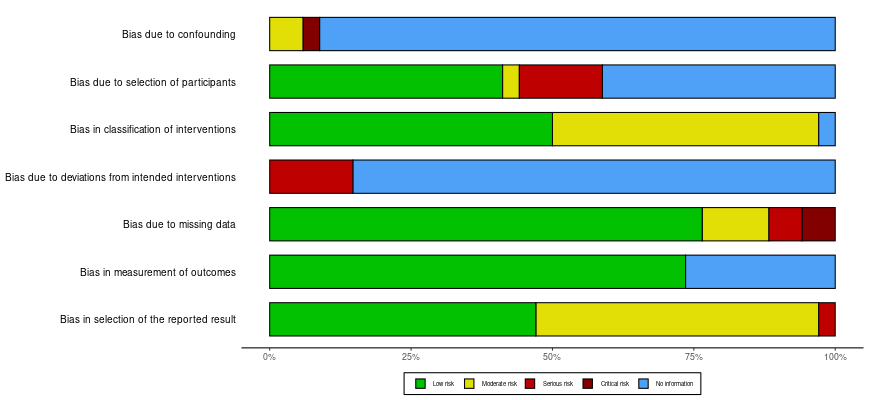


Risk of bias using ROB 2 for Randomized Controlled Trials (RCT) studies:


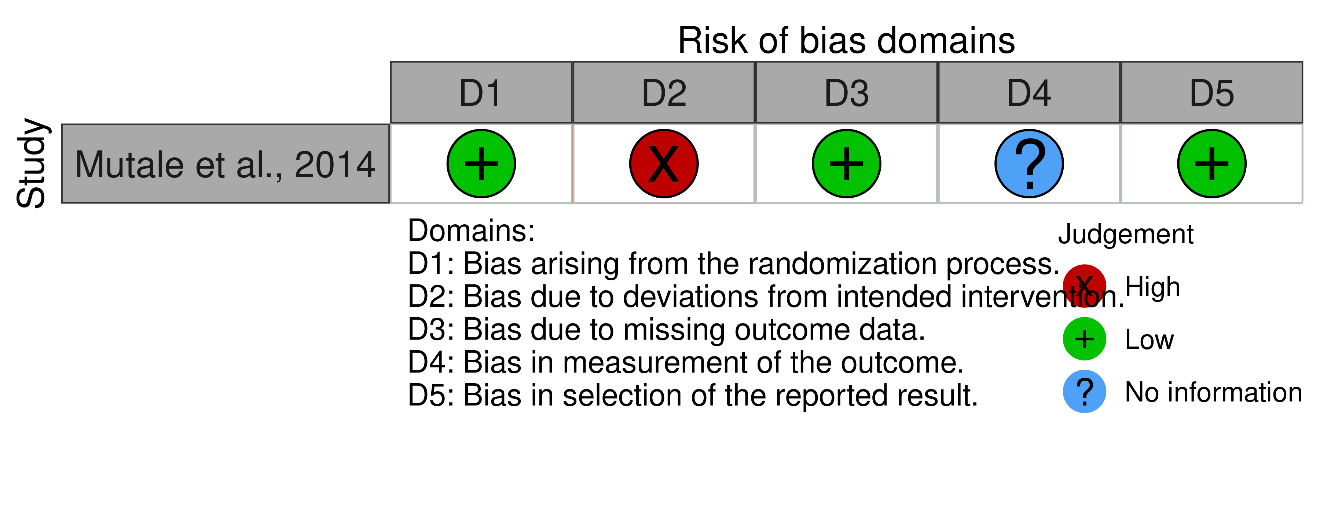

Supplement: Supplementary file 5 — Additional file 5. [file 12913_2022_7863_MOESM5_ESM.docx]
